# Supplementary material for: Fecal Microbiota and Associated Volatile Organic Compounds Distinguishing No-Adenoma from High-Risk Colon Adenoma Adults
Source: Metabolites. 2023 Jul 4;13(7):819. doi: 10.3390/metabo13070819 (PMC10383435; doi:10.3390/metabo13070819)
Supplement: Supplementary file 1 [file metabolites-13-00819-s001.zip › metabolites-2436900-supplementary.pdf]

Supplementary Information (SI)

Headspace SPME-GC-MS analysis of fecal VOCs: SPME fiber cleaning was performed for 30 minutes at 300 ^{o}C. The fiber was exposed to the stool sample within glass tubes for 30 minutes. It was then injected in the Agilent Single-Quadrupole GC/MS Instrument for 5 minutes, at 280 ^{o}C. GC-MS data processing for metabolome analysis (volatile identification and quantification) was carried out using the Agilent data analysis software. Raw GC-MS data files were automatically transferred to servers for storage and further analysis.

VOCs adsorbed on the 75μm CAR/PDMS SPME fiber were thermally injected into an electron impact (70 eV) quadrupole GC/MS (GC-7890B, MSD-5977B, Agilent Technologies, USA). The compounds were separated using SPB-624 capillary column (60 m x 250 μm, 1.4 μm film thickness) and helium (99.999%, Linde) as carrier gas (flow rate of 1.7 mL/min). The column remained at 35°C for 5 minutes, then increased to 180°C (at 4°C/min) for another 20 minutes. The chromatogram run time was 61.25 minutes. The temperatures of the MS transfer line, quadrupole, and ion source were 250, 150, and 230°C, respectively. Full-scan mode with 35-350 amu was selected. The NIST20 spectral library (≥ 80% match factor) allowed compound identification alongside an internal database of VOC standards. Data processing was performed using ChemStation software (Agilent, USA).

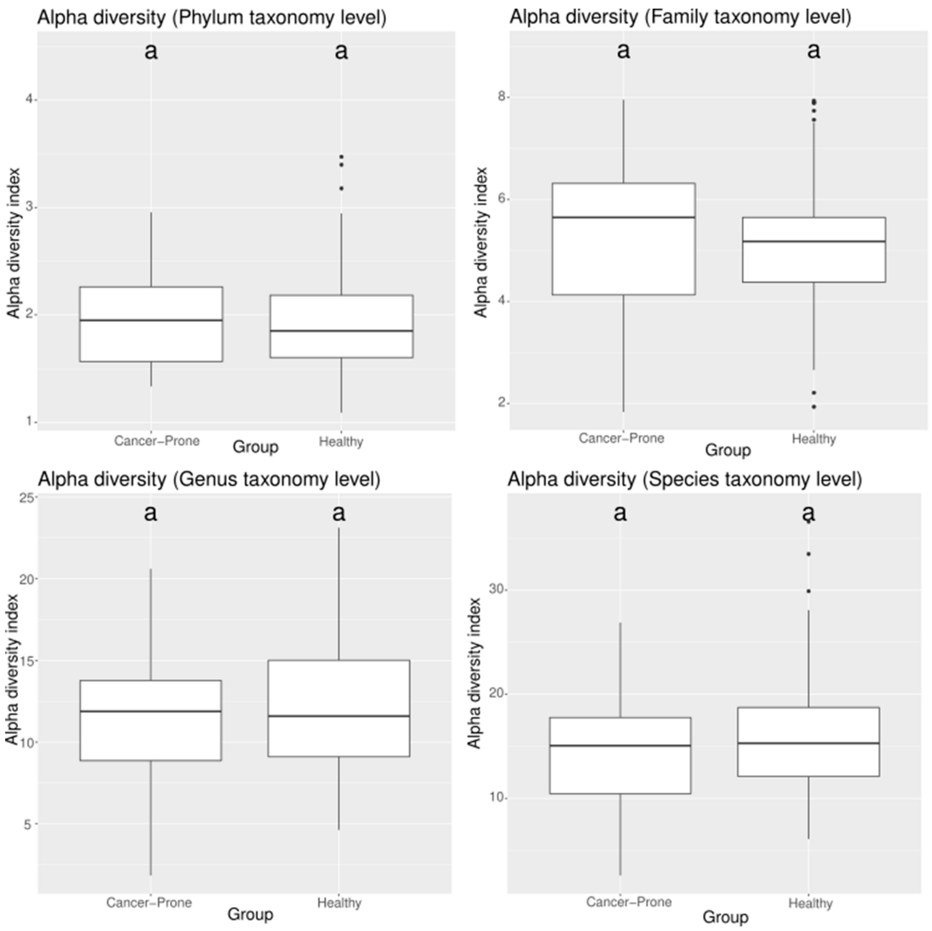

Figure S1. Alpha diversity plot. X-axis indicates the cancer-prone (HRA) and healthy (NA) groups and y-axis shows the alpha diversity index calculated for each taxonomic level with inverse Simpson. The mean of the alpha diversity found for each group is shown with the black horizontal line within each box. The results from the Tukey's honest significant difference (HSD) test are shown with the letters above each boxplot. Here, the letters are the same (a-a), which indicates that the alpha diversity index between the groups might not differ.

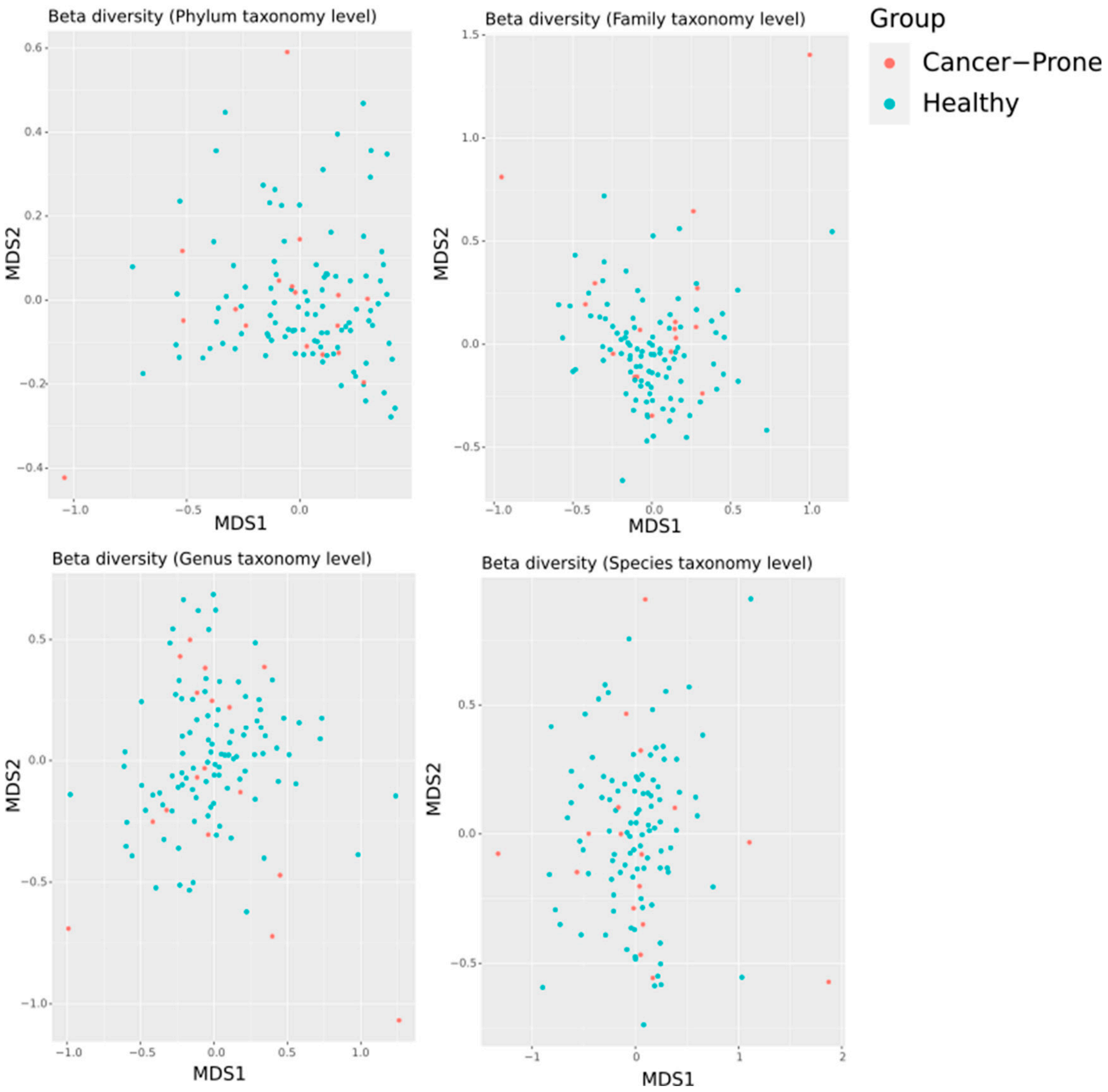

Figure S2. Multidimensional scaling (MDS) plot using UniFrac index, based on the relative abundance of the bacteria at the taxonomy level of phylum, family, genus, and species for samples between 17 NA and 100 HRA individuals after 16S metagenomic analysis. Orange and blue dots represent healthy (NA) and cancer-prone (HRA) individuals, respectively.

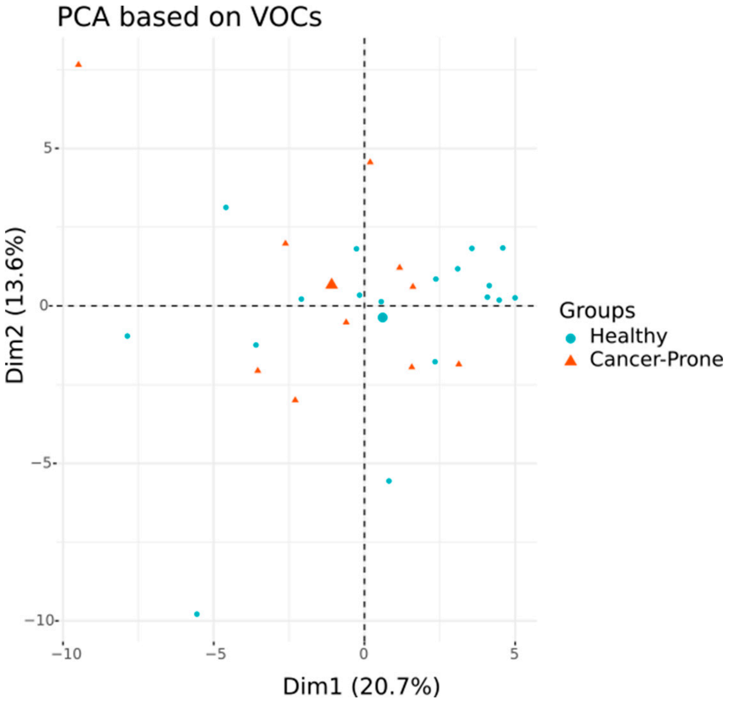

Figure S3. Principal component analysis (PCA) of fecal samples based on VOC composition. The plot is based on the normalized GC-MS values (Shapiro–Wilk normality test) of the 71 emitted volatiles for a total of 28 fecal samples classified in the NA group (18 subjects) and the HRA group (10 subjects). Orange triangles and blue dots represent healthy (NA) and cancer-prone (HRA) individuals, respectively. The big orange triangle and big blue dot represent the average healthy (NA) and cancer-prone (HRA) individuals, respectively.

Table S1. Statistically significant association between VOCs and subject groups. Information about the *p*-value, population size, and the mean value for each group is shown for all the statistically significant VOCs.

**VOCs**	***P*-value**	**HRA samples**	**NA samples**	**HRA mean**	**NA mean**
Methyl propionate	0,0466	10	18	0,7322	0,3475
Methyl butyrate	0,0370	10	18	2,2473	1,2484
Isobutyric acid	0,0393	10	18	0,0606	0,0402
Butyl methyl ketone (2-Hexanone)	0,0051	10	18	0,0541	0,0136
Ethyl acetone (2-Pentanone)	0,0452	10	18	0,1672	0,0956

Table S2. Statistically significant interactions between relative abundance of bacteria at the family level and VOCs. P-value, population size, mean relative abundance of FBFs, and mean value of VOCs for each group is also shown. The mean values were calculated based on the number of subjects used for the interaction test, that is, n=28 (NA=18 and HRA=10).

**FAMILY TAXONOMY LEVEL**							
**FBFs**	**VOC**	***P*-value of interaction**	**no.**	**Mean FBF value in HRA**	**Mean FBF value in NA**	**Mean VOC value in HRA**	**Mean VOC value in NA**
Acidaminococcaceae	D-Limonene	0,0333	28	2,1078	2,5298	0,0606	0,0402
	Isobutyric acid	0,0226	28	2,1078	2,5298	0,1755	0,1447
Bacteroidaceae	Isovaleraldehyde (3-Methylbutanal)	0,0188	28	13,6112	10,8085	0,0265	0,0649
	2-Methylbutyraldehyde (2-Methylbutanal)	0,0383	28	13,6112	10,8085	0,0561	0,0338
	Acetaldehyde	0,0097	28	13,6112	10,8085	0,1424	0,1516
	Acetic acid	0,0132	28	13,6112	10,8085	0,4298	0,2981
	Butanone	0,0159	28	13,6112	10,8085	0,1973	0,2447
	Isobutyraldehyde	0,0144	28	13,6112	10,8085	0,3416	0,2078
	Isobutyric acid	0,0314	28	13,6112	10,8085	2,2473	1,2484
	Methyl butyrate	0,0364	28	13,6112	10,8085	0,0606	0,0402
	Methyl cyclohexanecarboxylate	0,0483	28	13,6112	10,8085	0,0265	0,0265
	Propyl butyrate	0,0096	28	13,6112	10,8085	0,0860	0,0845
Coriobacteriaceae	Acetaldehyde	0,0230	28	3,2006	3,1246	0,0265	0,0649
	Dimethyl disulfide	0,0324	28	3,2006	3,1246	0,0036	0,0035

	Methacrolein	0,0333	28	3,2006	3,1246	0,0054	0,0027
	Methyl 4-methylvalerate	0,0300	28	3,2006	3,1246	0,5838	0,6092
	Propanal	0,0067	28	3,2006	3,1246	0,0462	0,0575
Enterobacteriaceae	Acetaldehyde	0,0486	28	1,2198	1,7108	0,0265	0,0649
	Methyl 4-methylvalerate	0,0126	28	1,2198	1,7108	0,0036	0,0035
	Propanal	0,0459	28	1,2198	1,7108	0,0462	0,0575
Erysipelotrichaceae	Acetaldehyde	0,0112	28	4,6086	2,5488	0,0265	0,0649
	Acetate	0,0370	28	4,6086	2,5488	0,1973	0,2447
	Dimethyl disulfide	0,0459	28	4,6086	2,5488	0,5838	0,6092
	Isobutyric acid	0,0286	28	4,6086	2,5488	0,0606	0,0402
	Isovalerate (Delphinic acid)	0,0482	28	4,6086	2,5488	0,1436	0,0892
	Styrene	0,0143	28	4,6086	2,5488	0,0045	0,0107
Eubacteriaceae	Diacetyl (2,3-Butanedione)	0,0047	28	1,7625	1,7783	0,1214	0,1014
	2-Methylbutyraldehyde (2-Methylbutanal)	0,0190	28	1,7625	1,7783	0,0561	0,0338
	Acetone	0,0277	28	1,7625	1,7783	0,0054	0,0027
	beta-Pinene	0,0358	28	1,7625	1,7783	0,1135	0,1520
	Ethyl acetone (2-Pentanone)	0,0277	28	1,7625	1,7783	0,4298	0,2981
	Isobutyraldehyde	0,0139	28	1,7625	1,7783	0,3416	0,2078
	Isovalerate (Delphinic acid)	0,0244	28	1,7625	1,7783	0,1672	0,0956

	Methacrolein	0,0120	28	1,7625	1,7783	0,0155	0,0197
	Methyl cyclohexanecarboxylate	0,0035	28	1,7625	1,7783	0,0860	0,0845
Lachnospiraceae	Dimethyl trisulfide	0,0073	28	26,3654	31,9963	0,0036	0,0035
	Methacrolein	0,0425	28	26,3654	31,9963	0,0054	0,0027
	Methyl 4-methylvalerate	0,0288	28	26,3654	31,9963	0,0462	0,0575
	Propanal	0,0362	28	26,3654	31,9963	0,2025	0,1480
	Propanal	0,0295	28	2,8706	0,0839	0,0036	0,0035
	Butyl methyl ketone (2-Hexanone)	0,0391	28	2,8706	0,0839	0,0541	0,0136
Peptostreptococcaceae	Decane	0,0280	28	1,5888	1,4099	0,0036	0,0035
	Isobutyrate	0,0437	28	1,5888	1,4099	0,0151	0,0125
	Methacrolein	0,0377	28	1,5888	1,4099	0,0054	0,0027
	Methyl cyclohexanecarboxylate	0,0413	28	1,5888	1,4099	0,0606	0,0402
	Propanal	0,0490	28	1,5888	1,4099	0,0034	0,0045
	Thiourea	0,0215	28	1,5888	1,4099	0,0860	0,0845
Porphyromonadaceae	Acetaldehyde	0,0497	28	1,7777	1,5011	0,0265	0,0649
	Methacrolein	0,0460	28	1,7777	1,5011	0,0162	0,0068
	Methanethiol (Methyl mercaptan)	0,0208	28	1,7777	1,5011	0,0054	0,0027
Prevotellaceae	Isovaleraldehyde (3-Methylbutanal)	0,0183	28	7,0502	2,8116	0,0561	0,0338
	2-Methylbutyraldehyde (2-Methylbutanal)	0,0167	28	7,0502	2,8116	0,4298	0,2981

	Acetic acid	0,0339	28	7,0502	2,8116	0,1973	0,2447
	Isobutyraldehyde	0,0104	28	7,0502	2,8116	0,3416	0,2078
Rikenellaceae	Acetaldehyde	0,0168	28	1,2148	0,8832	0,0265	0,0649
Ruminococcaceae	Acetaldehyde	0,0480	28	17,9953	22,1231	0,0265	0,0649
	Isovalerate (Delphinic acid)	0,0257	28	17,9953	22,1231	0,0036	0,0035
	P-Cresol	0,0392	28	17,9953	22,1231	0,1436	0,0892
	Propanal	0,0358	28	17,9953	22,1231	0,4585	0,4180
Streptococcaceae	Dimethyl trisulfide	0,0327	28	3,9527	2,3578	0,0162	0,0068
	Methanethiol (Methyl mercaptan)	0,0110	28	3,9527	2,3578	0,0036	0,0035
	Propanal	0,0160	28	3,9527	2,3578	0,2025	0,1480
	Sulcatone (6-Methyl-5-hepten-2-one)	0,0347	28	3,9527	2,3578	0,0286	0,0305
Veillonellaceae	D-Limonene	0,0342	28	3,7915	1,2730	0,1755	0,1447
Verrucomicrobiaceae	Butyl butyrate	0,0074	28	0,9157	1,7955	0,0265	0,0265
	Methyl cyclohexanecarboxylate	0,0265	28	0,9157	1,7955	0,1840	0,2770
	Propyl butyrate	0,0363	28	0,9157	1,7955	0,0072	0,0160
	Valeric acid (Pentanoic acid)	0,0089	28	0,9157	1,7955	0,0860	0,0845

Table S3. Statistically significant interactions between relative abundance of bacteria at the genus level and VOCs. P-value, population size, mean relative abundance of FBGs, and mean value of VOCs for each group is also shown. The mean values were calculated based on the number of subjects used for the interaction test, that is, n=28 (NA=18 and HRA=10).

**GENUS TAXONOMY LEVEL**							
**FBGs**	**VOC**	***P*-value of interaction**	**no.**	**Mean FBG value in HRA**	**Mean FBG value in NA**	**Mean VOC value in HRA**	**Mean VOC value in NA**
Akkermansia	Butyl butyrate	0,0497	28	0,9558	1,8369	0,5733	0,5769
	Butyrate (Butyric acid)	0,0075	28	0,9558	1,8369	0,0265	0,0265
	Methyl cyclohexanecarboxylate	0,0257	28	0,9558	1,8369	0,1840	0,2770
	Propyl butyrate	0,0347	28	0,9558	1,8369	0,0072	0,0160
	Valeric acid (Pentanoic acid)	0,0086	28	0,9558	1,8369	0,0860	0,0845
Alistipes	Acetaldehyde	0,0160	28	1,2508	0,9132	0,0265	0,0649
Anaerostipes	Dimethyl trisulfide	0,0485	28	1,0840	1,4862	0,2025	0,1480
Bacteroides	Isovaleraldehyde (3-Methylbutanal)	0,0172	28	13,8285	11,1753	0,0265	0,0649
	2-Methylbutyraldehyde	0,0362	28	13,8285	11,1753	0,0561	0,0338
	Acetaldehyde	0,0094	28	13,8285	11,1753	0,1424	0,1516
	Acetic acid	0,0117	28	13,8285	11,1753	0,4298	0,2981
	Butanone	0,0159	28	13,8285	11,1753	0,1973	0,2447
	Isobutyraldehyde	0,0128	28	13,8285	11,1753	0,3416	0,2078
	Isobutyric acid	0,0334	28	13,8285	11,1753	2,2473	1,2484

	Methyl butyrate	0,0369	28	13,8285	11,1753	0,0606	0,0402
	Methyl cyclohexanecarboxylate	0,0476	28	13,8285	11,1753	0,0265	0,0265
	Propyl butyrate	0,0104	28	13,8285	11,1753	0,0860	0,0845
Blautia	Propanal	0,0018	28	7,4343	8,6688	0,0036	0,0035
Clostridium IV	Acetic acid	0,0273	28	0,6284	1,3470	0,0054	0,0027
	Methacrolein	0,0196	28	0,6284	1,3470	0,1973	0,2447
Clostridium XlVa	Butanal	0,0012	28	2,3397	1,7719	0,0036	0,0035
	Propanal	0,0078	28	2,3397	1,7719	0,0175	0,0097
Clostridium XVIII	Decane	0,0490	28	1,6754	1,0891	0,0034	0,0045
Collinsella	Acetaldehyde	0,0409	28	2,2150	1,9674	0,0265	0,0649
	Dimethyl disulfide	0,0227	28	2,2150	1,9674	0,0036	0,0035
	Isobutyric acid	0,0138	28	2,2150	1,9674	0,0054	0,0027
	Methacrolein	0,0374	28	2,2150	1,9674	0,5838	0,6092
	Methyl 4-methylvalerate	0,0461	28	2,2150	1,9674	0,0606	0,0402
	Propanal	0,0165	28	2,2150	1,9674	0,0462	0,0575
	Acetaldehyde	0,0445	28	1,6960	1,9782	0,0265	0,0649
	Acetic acid	0,0239	28	1,6960	1,9782	0,1973	0,2447
	Active valeric acid (2-Methylbutanoic acid)	0,0479	28	1,6960	1,9782	2,2473	1,2484
	Butyrate (Butyric acid)	0,0043	28	1,6960	1,9782	0,0606	0,0402

Coprococcus	Isobutyric acid	0,0199	28	1,6960	1,9782	1,2991	0,7167
	Isovalerate (Delphinic acid)	0,0185	28	1,6960	1,9782	0,5733	0,5769
	Methyl butyrate	0,0257	28	1,6960	1,9782	0,1436	0,0892
	Methyl cyclohexanecarboxylate	0,0224	28	1,6960	1,9782	0,1151	0,0570
	Methyl valerate	0,0447	28	1,6960	1,9782	0,1840	0,2770
	Valeric acid (Pentanoic acid)	0,0255	28	1,6960	1,9782	0,0860	0,0845
Dialister	Propyl butyrate	0,0177	28	1,7181	0,1671	0,0265	0,0265
Dorea	Propanal	0,0154	28	1,5394	2,4240	0,0036	0,0035
Escherichia Shigella	Propanal	0,0160	28	0,4117	1,6384	0,0036	0,0035
Eubacterium	Isovaleraldehyde	0,0053	28	1,8049	1,8116	0,1214	0,1014
	Diacetyl (2,3-Butanedione)	0,0200	28	1,8049	1,8116	0,0561	0,0338
	2-Methylbutyraldehyde	0,0288	28	1,8049	1,8116	0,0054	0,0027
	Acetone	0,0389	28	1,8049	1,8116	0,1135	0,1520
	beta-Pinene	0,0306	28	1,8049	1,8116	0,4298	0,2981
	Ethyl acetone (2-Pentanone)	0,0158	28	1,8049	1,8116	0,3416	0,2078
	Isobutyraldehyde	0,0274	28	1,8049	1,8116	0,1672	0,0956
	Methacrolein	0,0124	28	1,8049	1,8116	0,0155	0,0197
	Methyl cyclohexanecarboxylate	0,0034	28	1,8049	1,8116	0,0860	0,0845
	Acetaldehyde	0,0084	28	6,2463	7,7564	0,0265	0,0649

Faecalibacterium	Dimethyl trisulfide	0,0417	28	6,2463	7,7564	0,0036	0,0035
	Furfural	0,0174	28	6,2463	7,7564	0,0024	0,0016
	P-Cresol	0,0133	28	6,2463	7,7564	0,0032	0,0083
	Propanal	0,0276	28	6,2463	7,7564	0,2025	0,1480
	trans-4-methyl-2-pentene	0,0256	28	6,2463	7,7564	0,4585	0,4180
Fusicatenibacter	Indole	0,0055	28	0,4490	1,3316	0,0036	0,0035
	P-Cresol	0,0340	28	0,4490	1,3316	0,4585	0,4180
	Propanal	0,0497	28	0,4490	1,3316	0,0365	0,0586
Gemmiger	Heptanal	0,0352	28	2,7335	3,0464	0,0118	0,0120
Holdemanella	Isovaleraldehyde	0,0202	28	1,2730	0,5205	0,0561	0,0338
	2-Heptanone	0,0305	28	1,2730	0,5205	0,4298	0,2981
	2-Methylbutyraldehyde	0,0297	28	1,2730	0,5205	0,3416	0,2078
	Ethyl acetone (2-Pentanone)	0,0427	28	1,2730	0,5205	0,1672	0,0956
	Isobutyraldehyde	0,0220	28	1,2730	0,5205	0,0462	0,0575
	Methyl 4-methylvalerate	0,0478	28	1,2730	0,5205	0,0182	0,0495
Lachnospiraceae incertae sedis	2-Octanone	0,0036	28	4,5114	5,1633	0,0036	0,0035
	Butanal	0,0365	28	4,5114	5,1633	0,0054	0,0027
	Dimethyl disulfide	0,0043	28	4,5114	5,1633	0,0175	0,0097
	Heptanal	0,0325	28	4,5114	5,1633	0,0185	0,0150

	Hexanal	0,0064	28	4,5114	5,1633	0,5838	0,6092
	Isobutyric acid	0,0079	28	4,5114	5,1633	0,0428	0,0223
	Isopropenyl methyl ketone	0,0429	28	4,5114	5,1633	0,0606	0,0402
	Methacrolein	0,0364	28	4,5114	5,1633	0,0118	0,0120
	Propanal	0,0103	28	4,5114	5,1633	0,0063	0,0040
Lactobacillus	Propanal	0,0316	28	2,9129	0,0861	0,0036	0,0035
	Butyl methyl ketone (2-Hexanone)	0,0381	28	2,9129	0,0861	0,0541	0,0136
Phascolarctobacterium	Dimethyl disulfide	0,0288	28	1,3843	2,1504	0,5838	0,6092
	D-Limonene	0,0365	28	1,3843	2,1504	0,1755	0,1447
Prevotella	Isovaleraldehyde	0,0160	28	6,2903	2,5045	0,0561	0,0338
	2-Methylbutyraldehyde	0,0142	28	6,2903	2,5045	0,4298	0,2981
	Acetic acid	0,0230	28	6,2903	2,5045	0,1973	0,2447
	Isobutyraldehyde	0,0102	28	6,2903	2,5045	0,3416	0,2078
Roseburia	Propanal	0,0239	28	2,1586	3,9047	0,0036	0,0035
Ruminococcus	Butyl methyl ketone (2-Hexanone)	0,0438	28	5,3763	7,3652	0,0541	0,0136
Ruminococcus2	Isovaleraldehyde	0,0007	28	2,8810	2,5121	0,0036	0,0035
	Hexanal	0,0466	28	2,8810	2,5121	0,0561	0,0338
	Isobutyraldehyde	0,0188	28	2,8810	2,5121	0,0924	0,0599
	Propanal	0,0339	28	2,8810	2,5121	0,4298	0,2981

	Propanol	0,0388	28	2,8810	2,5121	0,0428	0,0223
Streptococcus	Dimethyl trisulfide	0,0264	28	3,9984	2,2754	0,0162	0,0068
	Methanethiol (Methyl mercaptan)	0,0126	28	3,9984	2,2754	0,0036	0,0035
	Propanal	0,0145	28	3,9984	2,2754	0,2025	0,1480
	Sulcatone (6-Methyl-5-hepten-2-one)	0,0395	28	3,9984	2,2754	0,0286	0,0305
Veillonella	Propanal	0,0214	28	1,8410	0,4403	0,0036	0,0035
